# Promiscuous words

**DOI:** 10.1186/1742-9994-10-66

**Published:** 2013-11-08

**Authors:** Mark A Elgar, Therésa M Jones, Kathryn B McNamara

**Affiliations:** 1 Department of Zoology, University of Melbourne, Melbourne, VIC 3010, Australia; 2 Centre for Evolutionary Biology, The University of Western Australia, Crawley 6009, Australia

## Abstract

Promiscuity is frequently used to describe animal mating behaviour, and especially to describe multiple mating by females. Yet this use of the term is incorrect, perhaps reflecting an erroneous adoption of common language to pique reader interest. We evaluated the patterns of use and misuse of the word ‘promiscuity’ in a representative journal of animal behaviour. This survey highlights how inappropriately the term is used, and how it can conceal critical features of animal mating strategies with intriguing evolutionary significance. Further analysis of the scientific impact of papers identified by the term promiscuous or polyandrous revealed that the former were cited less frequently. We argue that using promiscuity to describe animal mating strategies is anthropomorphic, inaccurate, and potentially misleading. Consistent with other biological disciplines, the word promiscuity should be used to describe indiscriminate mating behaviour only, and that polygyny and polyandry should be used to describe male and female mating frequency respectively.

## Introduction

Promiscuity is frequently, but largely incorrectly used to describe animal mating behaviour, perhaps reflecting an erroneous adoption of common language to pique reader interest. According to *The Oxford English Dictionary*, promiscuous originally referred to repeated, indiscriminate actions: “That is without discrimination or method: confusedly mingled, indiscriminate (1605) … Of an agent or agency: making no distinctions: undiscriminating (1633) … casual, carelessly irregular (1837)” [1]. Promiscuity was used to describe human sexual activity in the 19^th^ Century, the essence (and costs) of which are colourfully observed in George Sala’s bawdy pantomime *Harlequin Prince Cherrytop* (1879): “Better frig, howe'er the mind it shocks, than from promiscuous … [fornication] … catch the pox” [2].

The term ‘promiscuity’ sneaked into the lexicon of evolutionary biology last century, particularly to describe mating behaviour e.g. [3-6] and is now widely entrenched (a *Web of Knowledge* (Thomson Reuters) search for ‘promiscu*’, limited to the fields of ‘Evolutionary Biology’, ‘Zoology’, ‘Behavioural Sciences’, and ‘Ecology’ returned over 700 publications). It is currently typically, although not exclusively [7], applied to describe *female* multiple mating or polyandry – the latter taking precedent [8].

Science often borrows words from common language: very early uses of the word promiscuous referred to surgical procedures [9], the use of barbiturates [10] and landscape management [11], and more recently molecular biologists use promiscuous to describe certain enzymes [12], gene regulators [13] and receptors [14] as promiscuous, precisely due to their non-specific nature. The use of these terms as scientific jargon draws on the general meaning of the word to highlight indiscriminating processes. This contrasts with its use as a descriptor for multiple mating behaviour, because the implied indiscriminating mate selection process is broadly wrong.

Females are rarely promiscuous, in the general meaning noted in the *Oxford English Dictionary*: the overwhelming evidence from diverse taxa confirms Darwin’s suggestion [15] that females are typically circumspect about their mates [16], accruing a variety of benefits from their discriminate mating [17,18], including with multiple partners [19]. In general, we expect females to remain choosy, irrespective of the number of mating partners, the exception being species in which there is cryptic female choice e.g. [20].

Promiscuous has been used as an umbrella term to include polyandry, polygyny, and polygynandry [21]. While it may be useful to use a single term to describe mating strategies in which males and females mate multiply (arguably, the modal animal mating behaviour), promiscuous is unhelpful because it conflates both the *nature* (discriminating or not) and *frequency* of mating. In contrast, the terms monogamy, polygyny, polyandry and polygynandry refer to frequency only. We highlight this issue by evaluating the patterns of use and misuse in the scientific literature of the word ‘promiscuity’ to describe female mating strategies.

## Use and misuse of promiscuous

We investigated whether polyandrous females were simultaneously described as promiscuous and exhibiting discriminating mate choice in papers published in a representative journal, *Animal Behaviour*. Drawing on papers published in the period 2000–2010, we identified those that contained ‘promiscuous’ (and its associated derivations) in either the abstract or main text. For each paper, we asked to which sex the term was applied (male, female or both), and whether the term was applied in a species in which pre-copulatory female choice had been experimentally demonstrated (either in the article itself or other published papers), or whether the authors inferred or suggested its presence in that species. Female mate choice is typically understood to mean a mating preference for different kinds of males [7,15,16], and is inferred from experiments or field observations showing that females prefer males according to the degree of exaggeration of secondary sexual characteristics e.g. [16-18]. We reduced the likelihood of misinterpretation of each paper by ensuring it was assessed independently by at least two readers. We confined our analysis to the term promiscuous because other descriptors of animal mating behaviour (such as polygynous, polyandrous and polygynandrous) do not make inferences about the nature of mating – whether either sex is discriminating or not – and thus are not at issue.

In total, 39 papers were evaluated (see Table 1). ‘Promiscuous’ was applied to females in 23 cases, males in 2, and in 14 cases the term was either applied to both sexes or the focal sex was ambiguous (significantly, such ambiguity is impossible with precise language, such as polyandry and polygyny). For papers in which ‘promiscuous’ was applied to females or both sexes (37 papers), female choice was demonstrated or suggested by the authors themselves in 18 instances, while in 15 cases there was no published evidence of female choice (four cases were excluded as the papers were theoretical reviews or meta-analyses). So, in over half of the instances, promiscuous is evidently used incorrectly, a proportion that is likely to be substantially underestimated: the absence of evidence of female choice in the remaining cases is not evidence that female preferences are absent.

**Table 1:** **Details of papers published in the journal *Animal Behaviour *** **that make reference to promiscuity**

**Title of paper**	**Publication details ( *Animal Behaviour *) year, volume, page numbers**	**Reference to promiscuity**					**Female choice?**^**1**^
		**Title**	**Abstract**	**Text**	**Key-words**	**Sex**	
Models of parent-offspring conflict 2. Promiscuity	1978, **26:**111–122	Yes	Yes	Yes	No	Female	—
Postcopulatory mate guarding delays promiscuous mating by female decorated crickets	1994, 48:1479–1481	Yes	—	Yes	No	Female	Yes
Mate sampling in a population of sand gobies	1997, **53:**267–276	No	Yes	Yes	No	Both	Yes
Behavioural correlates of monogamy in the noisy miner, *Manorina melanocephala*	1997, **54:**571–578	No	Yes	Yes	No	Female	No
Spawning success in the damselfish *Amblyglyphidodon leucogaster*: the influence of eggs in the nest	1998, **55:**651–664	No	Yes	Yes	No	Both	Yes
Behavioural aspects of the raccoon mating system: determinants of consortship success	1999, **57:**593–601	No	Yes	Yes	No	Female	Yes
Male mating behaviour and sperm production characteristics under varying sperm competition risk in guppies	1999, **58:**1001–1006	No	Yes	Yes	No	Female	Yes
Effects of body size and home range on access to mates and paternity in male bridled nailtail wallabies	1999, **58:**121–130	No	Yes	Yes	No	Both	Yes
Proximate factors associated with high levels of extra-consort fertilization in polygynous grey seals	1999, 58:527–535	No	Yes	Yes	No	Female	Yes
Sexual selection and the evolution of exclusive paternal care in arthropods	2000, **60:**559–567	No	Yes	Yes	No	Male	—
Lack of parasite-mediated sexual selection in a ladybird/sexually transmitted disease system	2002, **63:**131–141	No	Yes	Yes	No	Both	No
Sexual selection, multiple mating and paternity in grey mouse lemurs, *Microcebus murinus*	2002, **63:**259–268	No	Yes	Yes	No	Both	Yes
Genetic monogamy in Monteiro's hornbill, *Tockus monteiri*	2002, **63:**787–793	No	Yes	No	No	Female	No
The effects of sexual selection and life history on the genetic structure of redfronted lemur, *Eulemur fulvus rufus*, groups	2002, **64:**557–568	No	Yes	Yes	No	Female	No
Effects of repeated mating and polyandry on the fecundity, fertility and maternal behaviour of female earwigs, *Euborellia plebeja*	2003, **65:**205–214	No	Yes	No	No	Female	No
Spacing behaviour and its implications for the mating system of a precocial small mammal: an almost asocial cavy *Cavia magna*?	2003, **66:**225–238	No	Yes	Yes	No	Female	No
Behavioural defenses against sexually transmitted diseases in primates	2003, **66:**37–48	No	Yes	Yes	No	Female	—
Extrapair paternity in the common sandpiper, *Actitis hypoleucos*, revealed by DNA fingerprinting	2004, **67:**333–342	No	Yes	Yes	No	Female	No
Spacing pattern in a social group of stray cats: effects on male reproductive success	2004, **68:**175–180	No	Yes	Yes	No		No
Extrapair paternity and offspring immunocompetence in the reed bunting, *Emberiza schoeniclus*	2004, **68:**283–289	No	Yes	Yes	No	Female	Yes
Estimates of extreme sperm production: morphological and experimental evidence from reproductively promiscuous fairy-wrens (*Malurus*)	2004, **68:**541–550	Yes	Yes	Yes	No	Female	Yes
A pair choice test to identify female mating pattern relative to ovulation in longtailed macaques, *Macaca fascicularis*	2005, **70:**1283–1296	No	Yes	Yes	No	Female	No
Context-dependent male mating preferences for unfamiliar females	2005, **70:**1429–1437	No	Yes	Yes	No	Male	No
Social modulation of androgens in male vertebrates: meta-analyses of the challenge hypothesis	2006, **71:**265–277	No	Yes	Yes	No	Female	—
Number of mates and timing of mating affect offspring growth in the small marsupial *Antechinus agilis*	2006, **71:**289–297	No	Yes	Yes	No	Both	Yes
Variation in the cost to females of the sexual conflict over mating in the seed bug, *Lygaeus equestris*	2006, **72:**313–321	No	Yes	Yes	No	Both	No
The impact of lekking on the spatial variation in payoffs to resource-defending topi bulls, *Damaliscus lunatus*	2008, **75:**1229–1234	No	Yes	No	No	Female	Yes
Investment in eggs is influenced by male coloration in the ostrich, *Struthio camelus*	2009, **77:**1027–1032	No	Yes	Yes	No	Both	No
Male coloration reveals different components of immunocompetence in ostriches, *Struthio camelus*	2009, **77:**1033–1039	No	Yes	Yes	No	Both	Yes
Paternity assurance through frequent copulations in a wild passerine with intense sperm competition	2009, **77:**183–187	No	Yes	No	No	Female	No
Quantifying and comparing mating systems using normalized mutual entropy	2009, **77:**201–206	No	Yes	Yes	Yes	Both	—
Do male guppies distinguish virgin females from recently mated ones?	2009, **77:**425–431	No	Yes	No	No	Female	Yes
Another genetically promiscuous ‘polygynous’ mammal: mating system variation in *Neotoma fuscipes*	2009, **77:**449–455	Yes	Yes	Yes	Yes	Both	No
Male dominance rank and reproductive success in chimpanzees, *Pan troglodytes schweinfurthii*	2009, **77:**873–885	No	Yes	Yes	No	Female	Yes
Male feeding rate and extrapair paternity in the facultatively polygynous spotless starling	2009, **78:**1335–1341	No	Yes	Yes	No	Female	No
Male mate-searching strategies and female cues: how do male guppies find receptive females?	2010, **79:**1191–1197	No	Yes	Yes	No	Female	Yes
Plumage coloration, ejaculate quality and reproductive phenotype in the red-backed fairy-wren	2010, **79:**1239–1246	No	Yes	Yes	No	Female	Yes
Male aggression and sexual coercion in wild West African chimpanzees, *Pan troglodytes verus*	2010, **79:**333–342	No	Yes	No	No	Both	Yes
Sperm removal, ejaculation and their behavioural interaction in male cuttlefish in response to female mating history	2010, **79:**613–619	No	No	Yes	Yes	Both	No

^1^Not included for theoretical or review papers.

## Using promiscuity to titillate the reader?

Promiscuity as a term to describe animal mating behaviour is undoubtedly anthropomorphic, probably accounting for the frequency of its use, especially amongst the primate literature. The discipline does not tolerate other anthropomorphisms in biological science; for example, the term forced copulation is preferred over rape [22], and infanticide preferred over murder [23]. Promiscuity has pejorative and androcentric connotations [20] and is likely to be emotionally evocative [24], typically saved for the females of the species (Table 1): while polygynous males maximise their fitness by mating at the highest rate, females are described as promiscuous. Perhaps promiscuous is used in titles and abstracts precisely because it is titillating, the notion of indiscriminate mating tapping into latent social taboos.

**Figure 1:**
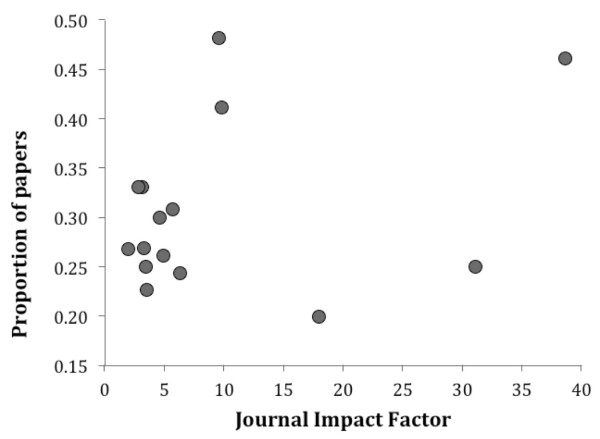
The proportion of ‘promiscuity’ papers in a journal was not associated with its Impact Factor (2012 Journal Citation Reports, Thomson Reuters) (Spearman’s ρ = 0.03, p > 0.9).

We explored the potential motivation for and consequences of using the term promiscuous by evaluating the citation metrics for papers retrieved by searches in *Web of Knowledge* (Thomson Reuters). We selected 15 journals and conducted two searches for each journal, using the terms (i) promiscuous OR promiscuity, and (ii) polyandrous OR polyandry (summarised in Table 2). We make the comparison with polyandry only because our previous analysis indicated that, in the vast majority of cases, promiscuity is used to describe female mating frequency (Table 1). Polygyny is widely understood to mean, based on the Greek etymology, multiple mating by *males*[24] and thus refers to an entirely different behaviour. Roughly a third of the papers included in the sample were identified by the term promiscuous or promiscuity in the title, abstract or key words. While this proportion ranged from 20–50% between journals, it was not correlated with the journal’s Impact Factor (Figure 1). Nevertheless, the mean number of citations of ‘polyandry’ papers (34 ± 7) per journal was marginally greater than that of ‘promiscuity’ papers (26 ± 4; Wilcoxon Sign-Rank test: p = 0.07), and the single-publication h-index of ‘polyandry’ papers (16 ± 2) was significantly higher than that of ‘promiscuity’ papers (9 ± 2; Wilcoxon Sign-Rank test: p < 0.0001) (Table 2). It is not clear whether this reflects an author’s publishing strategy, or that literature searches typically use the term polyandry over promiscuity.

**Table 2:** Characteristics of papers retrieved by the search term ‘promiscuity’ or ‘promiscuous’ and ‘polyandry’ and ‘polyandrous’ in 13 journals from 2000 to 31st July 2013

**Journal**	**Journal Impact Factor**^**1**^	**Papers retrieved by the search term ‘Promiscuity’ or ‘Promiscuous’**				**Papers retrieved by the search term ‘Polyandry’ or ‘Polyandrous’**				**% papers retrieved by promiscuous**^**3**^	**Δ cites**^**4**^
		**Papers**	**Most cites in a paper**	**Mean cites per paper**	**h-index**^**2**^	**Papers**	**Most cites in a paper**	**Mean cites per paper**	**h-index**^**2**^		
*American Naturalist*	4.55	9	205	43	7	21	118	24	13	30.0	19
*Animal Behaviour*	3.07	39	118	15	14	79	663	26	22	33.1	−11
*Behavioral Ecology*	3.22	21	54	14	9	57	67	16	19	26.9	−2
*Behavioral Ecology & Sociobiology*	2.75	40	50	15	15	81	51	18	23	33.1	−3
*Biology Letters*	3.35	6	35	10	4	18	29	10	8	25.0	0
*Current Biology*	9.49	13	37	9	5	14	146	32	7	48.1	−23
*Ecology Letters*	17.95	2	74	37	1	8	137	32	4	20.0	5
*Ethology*	1.95	11	37	12	5	30	120	16	12	26.8	−4.5
*Evolution*	4.86	23	103	23	12	65	113	28	28	26.1	−5
*J Evolutionary Biology*	3.48	17	62	13	9	58	86	21	22	22.7	−8
*Molecular Ecology*	6.28	20	90	24	13	62	421	31	27	24.4	−7
*Nature*	38.6	6	142	57	4	7	261	119	7	46.2	−62
*Proc Nat Acad Sci USA*	9.74	7	106	35	6	10	128	42	8	41.2	−7
*Proc Royal Society B*	5.68	37	106	39	24	83	128	32	33	30.8	7
*Science*	31.03	2	122	48	3	6	148	61	6	25.0	−13

(Note, *Frontiers in Zoology* is not included in the survey because no papers are retrieved by the search terms promiscuity or promiscuous, and only two papers were retrieved by the search terms polyandry or polyandrous).

^1^2012 Journal Citation Report®, ISI Web of Knowledge™.

^2^h-index calculated according to J.E. Hirsch in ISI Web of Knowledge™.

^3^Calculated as the number of papers retrieved by the search terms promiscuity or promiscuous, divided by the sum of the number of papers retrieved by the search terms promiscuity, promiscuous and polyandry or polyandrous.

^4^The mean cites per paper retrieved by the search terms promiscuity or promiscuous less the mean cites per paper retrieved by the search terms polyandry or polyandrous.

## Conclusions

Arguments over definitions can be tedious, but a cavalier use of borrowed words is unhelpful. Our surveys reveal a tendency to describe female rather than male mating strategies as promiscuous, despite the inherent contradiction in meaning. There was no evidence that journals of different standing publish more or fewer papers that use the term promiscuous, but authors searching for papers using the term promiscuous will generally retrieve lower impact publications.

Promiscuity has become so firmly entrenched in the literature as a synonym for polyandry that its accuracy is no longer questioned. But indiscriminately describing multiple-mating strategies as promiscuous conceals critical features of intriguing evolutionary significance. Indeed, records of truly promiscuous mating strategies, in which females (or males) mated indiscriminately would be remarkable, and predicted, for example, when the costs of mate choice are exorbitant. Like other emotionally evocative terms used to describe sexual behavior [25], promiscuity can be replaced with polyandry, polygyny and polygynandry, as appropriate – descriptive terms that are silent about the nature of mating, and devoid of sociological, psychological and moralistic connotations. Convention is no justification for imprecision, as our survey revealed: without evidence of indiscriminate mating behaviour, ‘promiscuity’ in evolutionary biology should be left well alone.

## Competing interests

The authors declare that they have no competing interests.

## Authors’ contributions

MAE, TMJ and KBM collated and prepared the data; MAE, TMJ and KBM analysed the data; MAE, TMJ and KBM drafted the manuscript. All authors read and approved the final manuscript.
